# Physical activity promotion by GPs: a cross-sectional survey in England

**DOI:** 10.3399/BJGPO.2021.0227

**Published:** 2022-07-27

**Authors:** Anna Lowe, Anna Myers, Helen Quirk, Jamie Blackshaw, Sabrina Palanee, Rob Copeland

**Affiliations:** 1 Advanced Wellbeing Research Centre, Sheffield Hallam University, Sheffield, UK; 2 Sheffield Hallam University, Sheffield, UK; 3 School of Health and Related Research, The University of Sheffield, Sheffield, UK; 4 Office for Health Improvement and Disparities, Department of Health and Social Care, London, UK; 5 King’s College Hospital NHS Foundation Trust, London, UK

**Keywords:** exercise, general practice, general practitioners, primary health care, public health

## Abstract

**Background:**

Physical activity (PA) contributes to the prevention and management of many health conditions. Primary care practitioners have an important role to play in supporting people to be physically active.

**Aim:**

This study had the following three aims: 1) to explore GPs‘ awareness and knowledge of the PA guidelines; 2) to assess GPs’ confidence in promoting PA; and 3) to explore factors that influence PA promotion among GPs.

**Design & setting:**

Cross-sectional survey, using secondary analysis.

**Method:**

UK-based GPs were invited to take part in an online survey in January 2021. Demographic questions were followed by nine multiple choice questions. Categorical data were analysed using descriptive statistics, and open-ended data were analysed using content analysis and inductive coding.

**Results:**

In total, 839 GPs based in England completed the survey. Most GP responders (98.9%) believed that PA was important, yet only 35.7% reported being at least ‘somewhat familiar’ with current PA guidance. Despite this, 74.1% of GPs reported feeling confident raising the topic of PA with their patients. Barriers included lack of time, perceptions of patient attitude and risk, language issues, and COVID-19. Key facilitators were identified and ‘Couch to 5k’ and the ‘parkrun practice’ initiatives were the most widely used support tools.

**Conclusion:**

GPs value PA yet well-known barriers exist to embedding promotion into primary care. As primary care reconfigures, there is an opportunity to embed PA into systems, services, and processes.

## How this fits in

PA is important in the prevention and management of health conditions, and healthcare professionals in primary care have an important role in supporting people to be physically active. The findings of this research indicate that GPs value PA and that most feel confident discussing PA with patients. It is not always easy for GPs to promote PA; there are many barriers, the most frequently cited of which is time constraints. As the landscape changes and primary care reconfigures, a joined-up approach to embedding PA promotion across primary care teams, systems, and services is needed.

## Introduction

Physical inactivity increases individual risk for all-cause mortality and is one of the leading causes of non-communicable disease in the world.^1^ It is well known that PA is effective in primary prevention, secondary prevention, and in the treatment of many long-term health conditions,^2^ yet inactivity rates in the UK are among the highest worldwide^3^ and follow an upwards trend. Tackling inactivity is complex; it involves multiple interacting factors that collectively influence individuals’ behaviours. Relevant domains include transport, built environment, societal norms and culture, sociopolitical influences, and psychological and biological factors.^4^

Health care is an important part of whole-system approaches to increasing PA.^5^ Primary care is recognised as having a central role in the continued national, strategic public health focus on increasing PA at a population level.^6,7^ This study refers specifically to the role of GPs within primary care settings. GPs are perceived as some of the most trusted professionals in society.^8^ They have privileged access into local communities and can reach those experiencing poorer health, older people, and those with lower socioeconomic status. This puts them in a unique position to support people to become more active.^3^ The average patient sees their GP >5 times per year,^9^ and around half of these contacts are related to management of long-term health conditions.^10^ These interactions have been identified as a critical opportunity to promote PA,^11,12^ and it has been reported that one in four people would be more active if advised by a GP or nurse.^13^ This is the rationale behind current national initiatives to embed PA into health care including the Moving Healthcare Professionals Programme.^14^

In 2016, a cross-sectional survey of GPs in England was undertaken^15^ to assess their knowledge, use, and confidence in relation to national Chief Medical Officers’ (CMO) PA guidance.^16^ The survey, published the following year, reported that most GPs (81%) were unfamiliar with the national PA guidance and that tools to assess PA were not routinely used in practice.^15^

The current study represents an important update on these initial findings. Five years on, the primary care system is reconfiguring into primary care networks (PCNs) and there are new roles such as link workers that, via social prescribing, offer a mechanism for PCNs to connect people to PA opportunities. These changes are occurring against the backdrop of COVID-19, which has created unprecedented pressures on primary care.^17^ The pandemic exacerbated existing health inequalities, with disadvantaged communities being disproportionately affected.^18^ It also highlighted population-level decreases in PA associated with periods of national lockdown, with certain groups, including older people, people from Black, Asian, or minority ethnic groups, and those with health conditions, being disproportionately affected.^19^

Collectively, these developments signal a substantial change in the context in which PA promotion in primary care occurs. This study offers a timely review of current practice and future opportunities.

### Aims and objectives

The primary aim of this study was to describe GPs’ experiences of promoting PA within the primary care setting. Objectives were to:

1. assess the perceived importance of PA among GPs in England;
2. assess GPs’ awareness of current CMO PA guidance;
3. assess GPs’ confidence in discussing PA with patients;
4. identify barriers to and facilitators of PA conversations with patients; and
5. assess the availability and appropriateness of tools that support the assessment and promotion of PA in primary care.

## Method

This study is a secondary analysis of existing survey data that was commissioned by The Office for Health Improvement and Disparities (OHID; formerly Public Health England) and collected by medeConnect Healthcare Insight, the market research division of the Doctors.net.uk (DNUK) group.^20^ This is the UK’s largest online professional network of doctors, providing information services to 245 274 doctors including GPs. Data were collected for 3 weeks between 8 and 29 January 2021 via an online omnibus questionnaire. GPs who logged in to DNUK during the 3-week window were invited to complete the questionnaire via a link that was visible on their personalised home page. Regional quotas were used to ensure proportionate representation. Two email reminders were sent during the study window to those who had opted-in to receive them. A small incentive (approximately 2 GBP of virtual points that are exchangeable for shopping vouchers or charitable donations) was provided on completion of all study questions.

The questionnaire was adapted from a previous survey.^15^ Questions were reviewed by an expert advisory panel including primary care clinicians, academics, and policy leads. Questionnaire design was coordinated by OHID and scripting was performed by medeConnect. The questionnaire consisted of nine multiple choice questions. Demographic information was also collected.

medeConnect collects and processes data in line with the Market Research Society code of conduct, and the legal and ethical framework of the British Healthcare Business Intelligence Association.^21,22^ All responders consented to their anonymised data being shared for research purposes.

### Data analysis

Categorical survey data were analysed using descriptive statistics. Statistical analysis was performed using IBM SPSS Statistics for Windows (version 24).^23^

The open-ended survey responses were used to help support and describe the quantitative data. The open-ended survey data were analysed in Excel using content analysis and inductive coding.^24^ A coding frame was devised inductively from the data, which involved manually assigning codes to the verbatim responses. These were cross-checked by a second member of the research team to determine agreement. Verbatim comments were extracted to illustrate the themes.

## Results

### Sample characteristics

At the time of this survey, DNUK had 12 374 active members (members who had used the website within the previous 90 days), and 10 410 members logged on to the website during the 3-week study period. Of those, 1305 GPs started the online questionnaire and 1005 completed it. For the purposes of this study, only responders based in England were included in analyses, which resulted in 166 participants being excluded, leaving a final sample size of 839 GPs.

Table 1 provides a breakdown of sample demographics. Responders were more likely to be male (53.6%), aged 36–55 years (75.5%), and GP partners or principals (52.2%). According to data from the General Medical Council, the proportion of males and females in the study sample was representative of England (males 52.9% and females 47.1%); however, GPs who were aged ≤35 years (28.7% in England) were underrepresented, and GPs aged 36–45 years (28.2% in England) and 46–55 years (20.7% in England) were overrepresented.^25^

**Table 1.** Demographic breakdown of sample (*N* = 839)

Category		*n*	%
Sex	Male	450	53.6
	Female	380	45.3
	Other	2	0.2
	Prefer not to say	7	0.8
Age, years	≤35	37	4.4
	36–45	354	42.2
	46–55	279	33.3
	≥56	169	20.1
Role	GP partner or principal	438	52.2
	Salaried GP	258	30.8
	Locum GP	142	16.9
	GP registrar	1	0.1

### Importance of PA, awareness of CMO guidance, and confidence to discuss PA with patients

**Figure 1.**
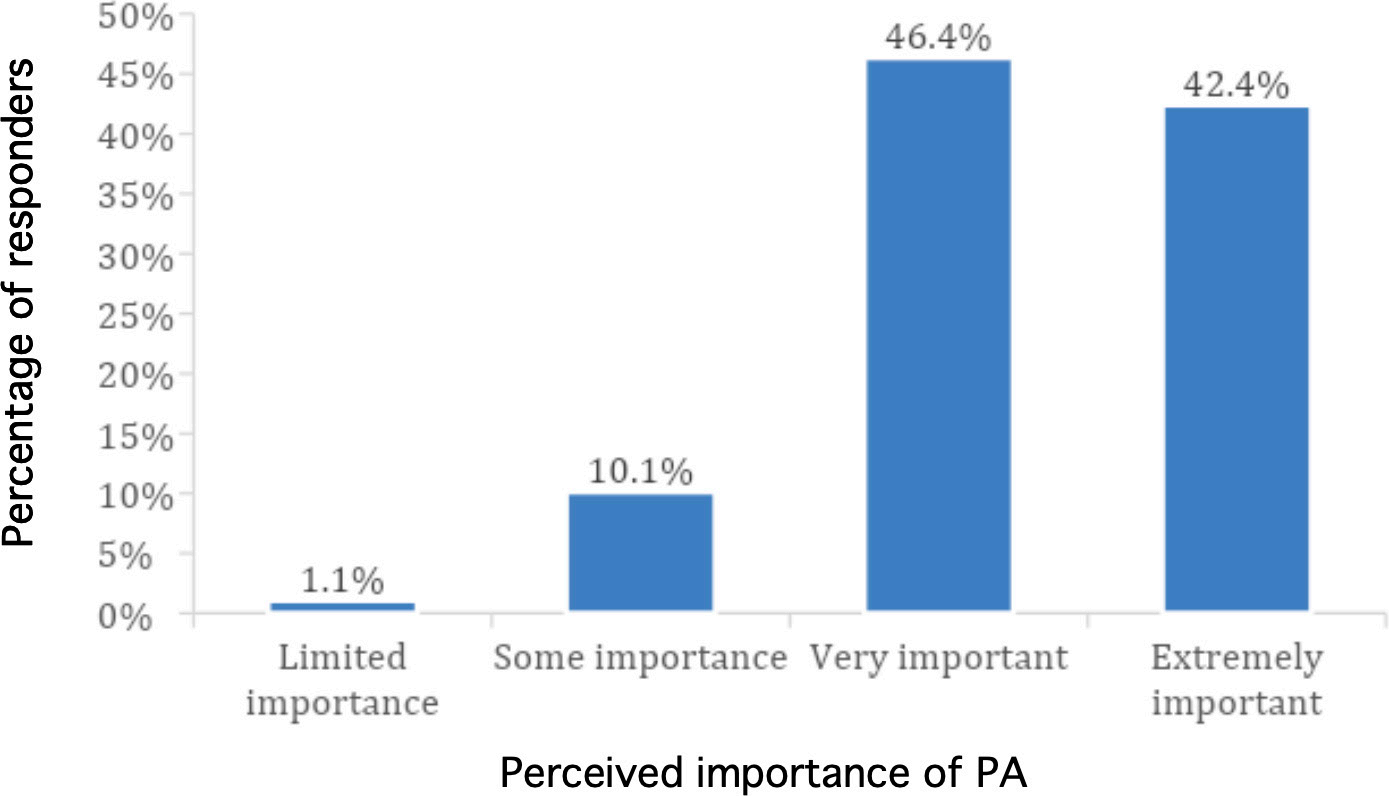
Perceived importance of physical activity (PA) for the prevention and management of health conditions

When asked about the importance of PA, most responders (98.9%) believed that PA was important (either of ‘some importance’ [10.1%], ‘very important’ [46.4%], or ‘extremely important’ [42.4%]) for the prevention and management of health conditions. As shown in Figure 1, only 1.1% believed PA was of limited importance.

**Figure 2.**
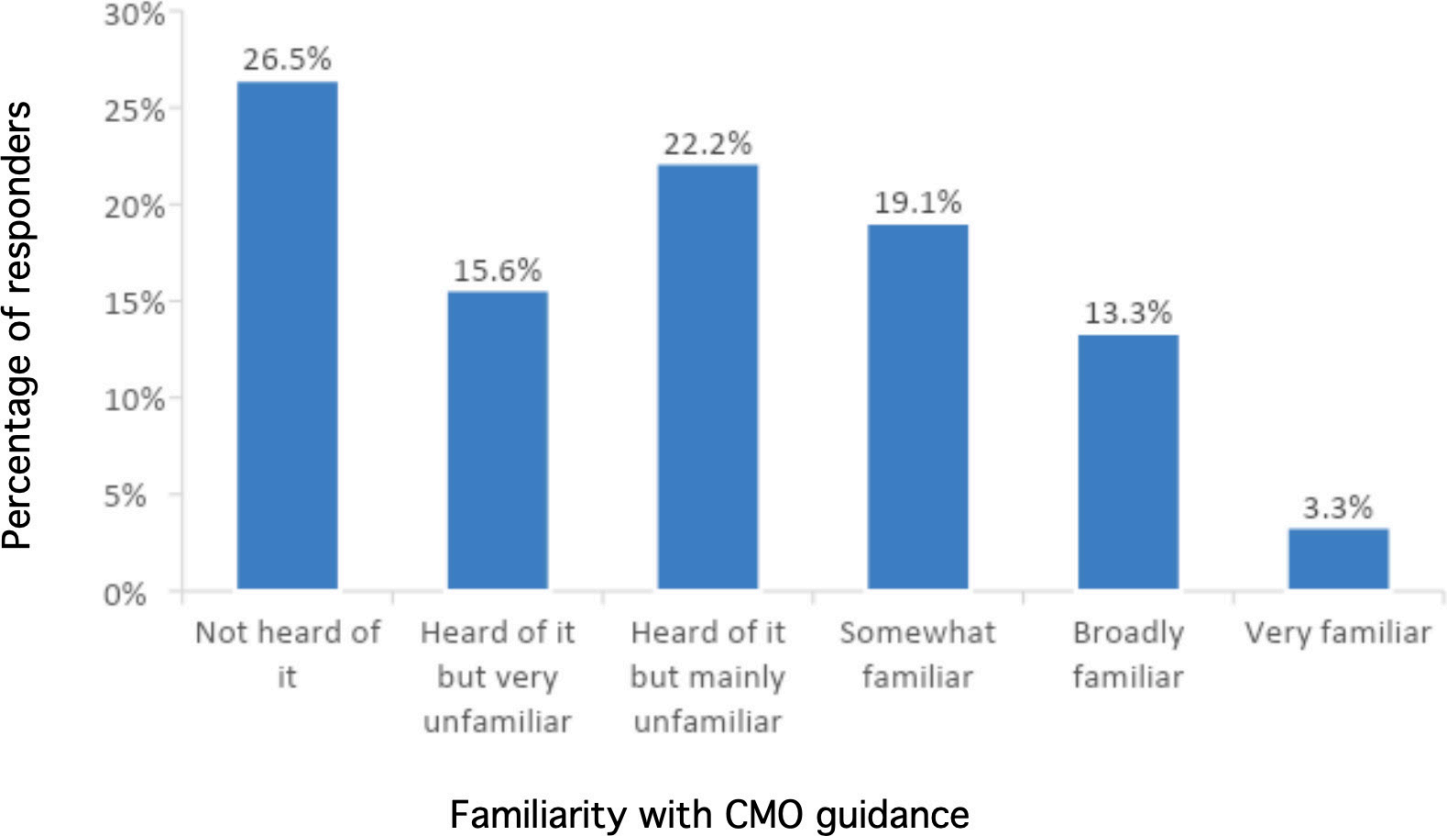
Familiarity with the Chief Medical Officers' physical activity guidance

GPs were asked about their awareness of the CMO PA guidance.^16^ Over one-quarter of responders had not heard of the guidance as illustrated in Figure 2.

**Figure 3.**
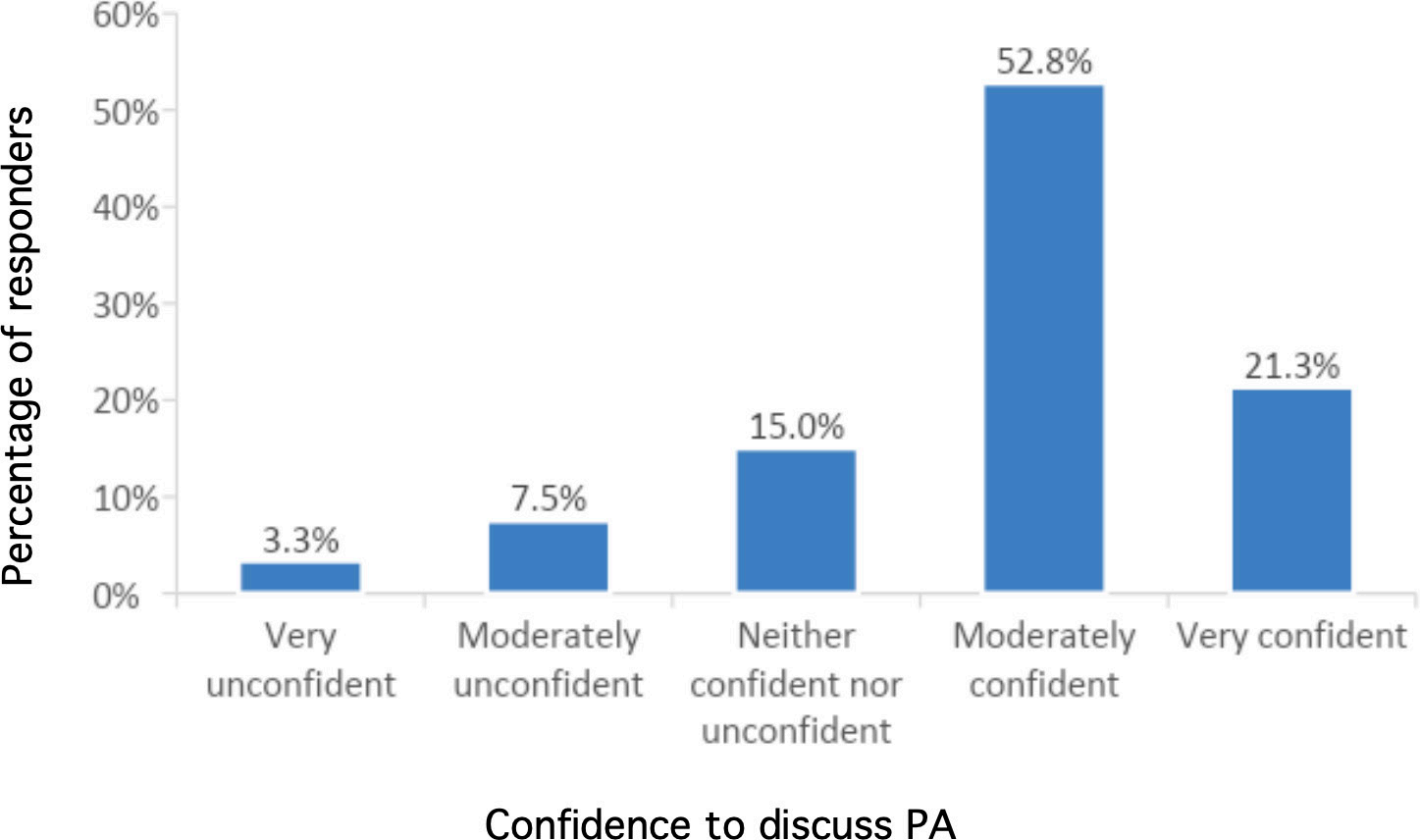
GPs’ confidence to discuss physical activity with their patients

When asked how confident GPs felt in discussing PA with patients, most participants (74.1%) reported being confident (either ‘moderately confident’ [52.8%] or ‘very confident’ [21.3%]) to discuss PA with their patients (Figure 3).

### Knowledge and awareness of PA tools, training, and campaigns

Responders reviewed a list of existing PA assessment tools (see Supplementary Figure S1). GPs had the greatest awareness of the General Practice Physical Activity Questionnaire (GPPAQ) (49.3%), but only 24.1% reported using it. Almost half (48.4%) of the responders were not aware of any of the PA measurement tools listed and 73.9% reported not using any.

When asked about existing PA training that GPs might have heard of and/or undertaken, most of the responders had not heard of (53.2%) or undertaken (73.2%) any of the PA training listed (see Supplementary Figure S2). ‘Using the General Practice Physical Activity Questionnaire (GPPAQ) in practice’ was the most widely known (25.5%) and completed (11.4%) but this was still a relatively small proportion. ‘Delivering brief interventions to encourage patient physical activity training’ was the second most widely known (18.5%) and it was undertaken by 8.0% of responders.

GPs were asked about their awareness of a range of PA campaigns and related tools. The most widely known tool was ‘Couch to 5k’ (known by 77.5% and used by 44.2%), followed by the ‘parkrun practice’ initiative (known by 66.4% and used by 32.2%). The third most known and used PA campaign or tool was ‘physical activity via social prescribing’ (known by 33.5% and used by 19.7%) (see Supplementary Figure S3).

GPs were asked about barriers and facilitators to effectively advising patients about PA (see Supplementary Figure S4). The top barriers were time available during consultations (77.8%), perceptions of patients’ attitude towards PA (62.1%), factors associated with the COVID-19 pandemic (for example, ability to prescribe safe PA; 54.2%), the patient's first language (54.0%), and concern by the patient about perceived risks of taking up PA (45.9%). The top five facilitators of PA promotion were the GP’s own PA behaviour/level (51.1%), awareness of local PA opportunities for patients (50.2%), trust in local PA deliverers (for example, physiotherapists, and fitness and leisure providers; 43.7%), the belief that social prescribers or local PA providers are best placed to provide advice (41.0%), and financial incentives (35.3%).

When asked about the perceived credibility of sources of PA information, the National Institute for Health and Care Excellence (NICE) website (70.6%) was reported as the most trusted source of information (see Supplementary Figure S5). Blogs were the least trusted sources of information across all the organisations and outlet types.

A follow-up question asked GPs what would help them to have more and/or better conversations with patients about PA. Having a better understanding of the local PA offer was the most highly cited (57.2%. This was followed by having better relationships and trust in local PA delivery (33.4%), availability of support tools (29.6%, and training (25.4%) (data not shown). As shown in Table 2, content analysis of the optional open-text response to this question indicated a perception that more time in consultations would enable better quality PA conversations within clinical practice (75% of the 131 responders who provided an open-text comment reported ‘more consultation time’ would help them have PA conversations with patients) as exemplified in the following quote:

*‘A lot more time in consultations would help. We are struggling with the normal workload and the COVID pandemic plus the massive vaccination programme is making discussions about exercise a low priority.’* (GP280)

**Table 2.** Overview of codes from content analysis of open-text answers

Codes: what would help GPs to have more, or better quality physical activity conversations within clinical practice?	Frequency code was reported, *n*	Example responses
More consultation time	98	*'Seriously, we have no time to do any of this! We need time to actually do our medical jobs. If people really need to be advised how to exercise, why does that responsibility (like everything else!!!) fall to GPs?!!!'* (GP495)
Referrals — other or better resources available and improved links to local services	22	*'The process for referral to local providers and the funding or discounting of these keeps changing. Consistent easy pathways would help.'* (GP375)
Not part of GP job role — others are better suited	12	*'I think this is the job of public health campaigns.'* GP472)*'In fact I feel that HCA [healthcare assistants] would be better place[d] to do that during chronic disease review.'* (GP110)*'No time really, there should be a physical activity advocate to refer to.'* (GP575)
COVID or lockdown	5	*'A lot more time in consultations would help. We are struggling with the normal workload and the COVID pandemic plus the massive vaccination programme is making discussions about exercise a low priority.'* (GP280)
Financial; incentive or funding to do it	5	*'Time and resources and payment for time at work to do this.'* (GP832)*'The process for referral to local providers and the funding or discounting of these keeps changing. Consistent easy pathways would help.'* (GP375)
More GPs	3	*'About 10 000 more GPs.'* (GP492)'*other staff to do it'* (GP285)
Reduced workload	3	*'Increased consultation time.Reduced workload.'* (GP400)*'More time currently just coping with normal workload.'* (GP783)
Better patient attitudes and/or willingness towards exercise	3	*'More time in consultation and patient willingness.'* (GP110*)* *'More time and better attitudes towards exercise.'* (GP156)
Wider cultural change needed	3	*'Widespread cultural and societal changes. GP input is a drop in the ocean.'* (GP621)
Funding for patients	1	*'Bursaries or funded opportunities for patients.'* (GP788)
Change in GP model	1	*'Complete change in GP model.'* (GP782)
More time for my own health	1	*'More time with patients and more time for my own health.'* (GP227)
GP training	1	*'Motivational interview training I did was helpful (not the one specified but one arranged at CCG level).'* (GP264)

CCG = clinical commissioning group.

## Discussion

### Summary

PA is important for the prevention and management of numerous health conditions. Primary care settings have an important role to play in supporting people to be active as part of a whole-system approach.^5^ This study aimed to describe GPs’ experiences of promoting PA within the primary care setting.

Findings show that almost all GP responders (98.9%) believed that PA is important in the prevention and management of health conditions. Although many were not familiar with the CMO guidance on PA, the majority reported feeling confident raising the topic of PA with their patients. Awareness and use of PA tools to assess a patient’s PA varied; the most widely known and used tool was the GPPAQ. Many of the other available tools were not known to GPs. The most widely cited barrier to discussing PA with patients was a lack of time; this was underscored through analysis of open-text responses. The most common facilitators were GPs’ own PA behaviour and awareness of local PA opportunities. Awareness of, and engagement with, PA-related training was generally low. With regard to PA campaigns and support tools, ‘Couch to 5k’ and the ‘parkrun practice’ initiatives were the most widely known and used. Finally, GP responders perceived the NICE website to be the most trusted source of information, and blogs were perceived as the least trusted.

### Strengths and limitations

This survey accessed a large sample of GPs as part of an omnibus survey that was not explicitly about PA. This may reduce the bias associated with attracting only PA advocates. The sample was self-selected, in that GPs opted-in to the omnibus survey. It was noted that GPs in training (GP registrars) were underrepresented with a predominance of older, more experienced GP responders. Data collection was undertaken in January 2021 in the midst of the COVID-19 pandemic, which might have impacted on recruitment and responses.

### Comparison with existing literature

The findings build on those of Chatterjee *et al*,^15^ providing an update on GPs’ perspectives on PA promotion within the primary care context. Comparisons must be interpreted with caution, however: despite using similar methods, the wording of the questions was changed along with response options, making direct comparison inappropriate. Instead noteworthy trends are highlighted.

The findings of the present study suggest that 35.7% of responders were at least somewhat familiar with the CMO guidance on PA, which is a greater proportion of responders than in 2017 when only 20% were familiar. The proportion of GPs who reported feeling confident to discuss PA with patients is also greater than reported in the 2017 survey and the proportion feeling unconfident decreased. Previous literature has indicated that primary care providers might lack knowledge and confidence in discussing and promoting PA among patients.^26–28^ However, the current findings suggest that, while familiarity with the CMO PA guidance remains low, three-quarters of GPs felt confident to discuss PA with patients.

The findings regarding use of GPPAQ to assess PA in primary care mirror those of Chatterjee *et al;* ^15^ it remains the best-known tool (49%) but use remains relatively low (24%). This warrants further exploration; pragmatic, integrated assessment tools could facilitate conversations about PA and help to make it ‘part of the process’ within clinical practice. However, despite being launched in 2006, findings suggest that the GPPAQ is not part of routine practice for many GPs.

When asked about specific PA tools and campaigns that were used in practice ‘Couch to 5k’ was the best known (78%) followed by the ‘parkrun practice’ initiative (66%). This suggests significant reach of these initiatives, perhaps explained by the partnerships these programmes have with NHS and Royal College of General Practitioners, respectively. It is surprising that awareness of social prescribing was not higher, with only 36% of GPs reporting that they were aware of it. This may reflect the fact that it is relatively early in the implementation of social prescribing and the extent to which it is embedded varies regionally.

Lack of time was the most reported barrier to discussing PA with patients, a finding that reflects existing literature.^29,30^ In the UK, the GP workload is the highest it has ever been, and existing time constraints have been compounded by increased pressure on primary care owing to COVID-19.^17^ As the primary care system reconfigures, there is opportunity to embed PA across primary care. This notion speaks to the growing body of evidence that engaging the whole system is the only viable way to create meaningful change in population PA levels, as noted by McAuley *et al*:^31^

*'Doctors can be trained to deliver opportunistic advice on behaviour change, including exercise, but that doesn't necessarily change population levels of inactivity if efforts are sporadic, confined to motivated practitioners, or isolated from the societal factors that cue inactive choices.'*

Another key barrier to PA promotion that was frequently cited by GPs was the patient's first language not being English. People who cannot speak English well are more likely to be in poor health and experience a more rapid decline of good health by age.^32^ Nobles *et al* ^33^ highlighted the need for culturally tailored language when communicating PA messages to underserved communities. This finding warrants further investigation owing to its potential to further exacerbate health inequalities.

Almost half of GP responders (45.9%) cited that concern from the patient about perceived risks of taking up PA acts as a barrier to advising patients on PA. This underscores the importance of the role of primary care clinicians in helping patients to interpret risk appropriately and in facilitating conversations that help patients to balance the inherent risks of physical inactivity with risks associated with increases in PA. Such conversations will be helped by the recent consensus statement confirming the low risk associated with PA for most people living with long-term conditions from Reid *et al*.^34^

### Implications for research and practice

The headline finding that GPs value PA is positive. There are opportunities to explore how PA promotion can be made easier for GPs in the face of ongoing barriers. In addition to well-known barriers,^28,29^ the present research also suggests additional barriers include communicating effectively with people who cannot speak English. If communication issues prevent GPs from promoting PA to people known to experience worse health, it has the potential to widen existing health inequalities and should be a priority for further research and action.

The findings suggest that promotion of PA in primary care could be facilitated through the design of tools that are intuitive, integrate with existing systems, and enable quick assessment of PA. This could be helpfully explored, as could the development of appropriate training of GPs in the promotion of PA. The findings also illustrate that community PA offers, such as ‘parkrun practice’ and ‘Couch to 5k’ are widely used by GPs. GPs clearly feel the need to know more about local offers; however, as social prescribing is scaled-up and link workers are embedded, it may enable GPs to reinforce critical messaging and then hand over to team members who have current community connections.

GPs need to be supported by other members of the primary care team and the wider community, and enabled by systems and processes within primary care if they are to be effective in promoting PA. The individual action of GPs could be enhanced by stronger and closer connections between primary care and local authorities, PA providers, public health teams, Active Partnerships, integrated care systems, and other key parts of the broader system. As the landscape of primary care changes, a joined-up approach to embedding PA promotion across primary care is needed.

## Data

The datasets generated during and/or analysed during the current study are available from the corresponding author on reasonable request.
